# The regulation of mitochondrial transcription factor A (Tfam) expression during skeletal muscle cell differentiation

**DOI:** 10.1042/BSR20150073

**Published:** 2015-06-25

**Authors:** Melania Collu-Marchese, Michael Shuen, Marion Pauly, Ayesha Saleem, David A. Hood

**Affiliations:** * School of Kinesiology and Health Science, Muscle Health Research Centre, York University, Toronto, Ontario, Canada M3J 1P3

**Keywords:** adenosine 5′-phosphate (AMP) kinase, mitochondrial biogenesis, messenger ribonucleic acid (mRNA) stability, myogenesis, transcription

## Abstract

The ATP demand required for muscle development is accommodated by elevations in mitochondrial biogenesis, through the co-ordinated activities of the nuclear and mitochondrial genomes. The most important transcriptional activator of the mitochondrial genome is mitochondrial transcription factor A (Tfam); however, the regulation of Tfam expression during muscle differentiation is not known. Thus, we measured Tfam mRNA levels, mRNA stability, protein expression and localization and Tfam transcription during the progression of muscle differentiation. Parallel 2-fold increases in Tfam protein and mRNA were observed, corresponding with 2–3-fold increases in mitochondrial content. Transcriptional activity of a 2051 bp promoter increased during this differentiation period and this was accompanied by a 3-fold greater Tfam mRNA stabilization. Interestingly, truncations of the promoter at 1706 bp, 978 bp and 393 bp promoter all exhibited 2–3-fold higher transcriptional activity than the 2051 bp construct, indicating the presence of negative regulatory elements within the distal 350 bp of the promoter. Activation of AMP kinase augmented Tfam transcription within the proximal promoter, suggesting the presence of binding sites for transcription factors that are responsive to cellular energy state. During differentiation, the accumulating Tfam protein was progressively distributed to the mitochondrial matrix where it augmented the expression of mtDNA and COX (cytochrome *c* oxidase) subunit I, an mtDNA gene product. Our data suggest that, during muscle differentiation, Tfam protein levels are regulated by the availability of Tfam mRNA, which is controlled by both transcription and mRNA stability. Changes in energy state and Tfam localization also affect Tfam expression and action in differentiating myotubes.

## Abstract (precis)

The present study highlights the transcriptional and post-transcriptional regulation of mitochondrial transcription factor A (Tfam) during myogenesis and the relationship this has with the increase in mitochondrial content during differentiation.

## INTRODUCTION

The fusion of myoblasts into myotubes is vital for skeletal muscle development during embryogenesis and for the maintenance of muscle fibres throughout the lifespan [1]. Progression into immature muscle fibres is accompanied by an increase in mitochondrial content to provide the cell with the adequate ATP levels necessary for proper skeletal muscle function. Skeletal muscle cells devoid of mitochondria lose the ability to differentiate, implicating the importance of mitochondrial biogenesis for proper cellular development. The rapid induction of the organelle's mitochondrial content during differentiation requires a collaborative regulation of both the nuclear and the mitochondrial genomes. Mitochondrial transcription factor A (Tfam) is a nuclear-encoded protein that promotes the expression of the 13 proteins, 22 tRNAs and 2 rRNAs expressed by mtDNA [2,3]. Despite the fact that the nucleus encodes the vast majority of the structural and functional proteins which comprise the organelle, a parallel up-regulation in the transcription of these mitochondrial genes is mandatory for mitochondrial biogenesis. Following import into the mitochondria, Tfam appears to have a high affinity for the light- and heavy-strand promoters of the mitochondrial genome [4,5]. mtDNA transcription is enhanced when Tfam binds to mtDNA along with mitochondrial RNA polymerase and either mitochondrial transcription factor B1 (TFB1M) or B2 (TFB2M) [6]. Because transcription is proposed to be coupled with replication, Tfam is also important in maintaining mtDNA copy number. Consistent with this notion, Tfam homozygous knockout mice have depleted mtDNA and are not viable beyond embryonic development due to a severe respiratory chain deficiency, whereas heterozygous knockout mice have a reduced mtDNA copy number [7]. Thus, Tfam is clearly important in regulating mitochondrial biogenesis and cellular metabolism within the cell.

The induction of nuclear-encoded mitochondrial proteins such as Tfam often occurs via the activation of multiple serine-threonine kinases in skeletal muscle. AMP-activated protein kinase (AMPK) is activated in muscle by increases in the AMP:ATP ratio. This kinase specifically enhances the transcription and activity of peroxisome proliferator-activated receptor-γ coactivator 1α (PGC-1α), an important regulator of mitochondrial biogenesis [8–10]. AMPK is also important in promoting the expression of nuclear-encoded mitochondrial proteins through the activation of transcription factors [8]. In addition, a study using mice with constitutively active AMPK found increases in Tfam mRNA expression [11]. Thus, AMPK activation is likely to be important for Tfam gene expression, but its role during muscle development is not known. Indeed, there is limited understanding about the factors which regulate the expression of Tfam under physiological condition in which mitochondrial biogenesis is accelerated. Thus, the purpose of the present study was to address this question by examining the pathway of Tfam expression, from transcription to localization, during mitochondrial biogenesis induced by muscle differentiation. We hypothesized that Tfam expression would increase during muscle cell differentiation and that this would be controlled largely by the availability of mRNA.

## EXPERIMENTAL

### Cell culture

C_2_C_12_ myoblasts were cultured on 100 mM gelatin-coated plastic culture plates containing Dulbecco's modified Eagle's medium [DMEM; supplemented with 10% FBS and 1% penicillin/streptomycin (P/S)]. Cells were incubated in conditions of 5% CO_2_ at 37°C. Once reaching 90% confluence, differentiation was induced by switching to DMEM supplemented with 5% heat-inactivated horse serum and 1% P/S. For experiments utilizing 5-aminoimidazole-4-carboxamide ribonucleotide (AICAR), cells were treated for 24 h with either water or AICAR (1 mM), to activate AMP kinase [12].

### Cytochrome *c* oxidase activity

C_2_C_12_ myoblasts were washed and scraped with ice-cold Dulbecco's PBS (DPBS, Sigma–Aldrich). After centrifugation at 3000 ***g*** for 5 min, pellets were resuspended in 100 mM Na-KPO_4_/2 mM EDTA (pH 7.2), sonicated on ice (3×3s), frozen in liquid N_2_ and then thawed. Samples were then centrifuged at 16000 ***g*** for 5 min at 4°C and the supernatants were mixed with test solutions containing reduced cytochrome *c*. Enzyme activity was determined by measuring the maximal rate of oxidation of reduced cytochrome *c* using the change in absorbance at 550 nm on a Bio-Tek Synergy HT microplate reader.

### Protein extraction and immunoblotting

C_2_C_12_ myoblasts were washed with PBS then scraped with a passive lysis buffer supplemented with protease inhibitors to generate whole cell lysates. Cytoplasmic and mitochondrial fractions were also isolated from cultured cells at 0, 2 and 4 days of differentiation and were prepared using the Mitochondria or the Nuclear Isolation Kits (Pierce). Equal amounts of total protein (35–70 μg) were mixed with loading buffer and separated on SDS/PAGE (10%, 12% or 15% gels). Gels were then transferred on to nitrocellulose membranes and blocked in for 1 h in 5% skim milk-1× TBST (Tris-buffered saline and Tween-20). Blots were incubated overnight at 4°C with antibodies (diluted in blocking buffer) specific for Tfam (1:1000, donated by Dr H. Inagaki, Research Institute of Nagoya, Japan), cytochrome *c* oxidase (COX) IV (1:1000, Calbiochem), cytochrome *c* (1:750, made in-house), Sp1 (1:200, Santa Cruz), p65 (1:1000, Santa Cruz), p-AMPK (1:500, Cell Signaling), human antigen R (HuR) (1:4000, Santa Cruz), CUG triplet repeat RNA-binding protein 1 (CuGBP1) (1:1000, Santa Cruz), voltage-dependent anion channel (VDAC), GAPDH (glyceraldehyde-3-phosphate dehydrogenase; 1:5000, Abcam) and KH type splicing-regulatory protein (KSRP) (1 ml of pre-diluted antibody serum donated by Dr B. Jasmin, University of Ottawa). After incubation, the blots were washed (3×5 min) in TBST and probed with the appropriate anti-mouse (α-Tubulin, COX IV), anti-rabbit (cytochrome *c*, Tfam) or horseradish peroxidase-conjugated secondary antibody at room temperature for 1 h. α-Tubulin was used as a loading control for cell lysate and cytoplasmic fractions. The membrane was then subjected to enhanced chemiluminescence (Amersham Pharmacia Biotech) and exposed to film. Signals were quantified with Sigma Scan Pro.

### RNA isolation and Tfam mRNA quantification during differentiation using RT-PCR

C_2_C_12_ myoblasts were harvested and total RNA was extracted using TRIZOL (TRIzol® trademarked product). Total RNA was resuspended in diethyl pyrocarbonate-treated sterile H_2_O. RNA purity and concentrations were determined spectrophotometrically at 260- and 280-nm. mRNA levels were measured using reverse transcription (RT)-PCR for Tfam at each time point. RT was performed using Superscript II Reverse Transcriptase (Invitrogen) according to the instructions from the manufacturer. Forward and reverse primers (Tfam: Forward 5′-AAGGGAATGGGAAAGGTAGA-3′/Reverse 5′-AACAGGACATGGAAAGCAGAT-3′; β-actin: Forward 5′-TGTGACGTTGACATCCGTAA-3′/Reverse 5'-GCTAGGAGCCAGAGCAGTAA-3') were used. PCR products were analysed on Ethidium Bromide-stained agarose gels and expected size products were quantified using SigmaScan Pro (version 5) software. The quantification of all target genes was corrected using the internal control β-actin.

### Mouse Tfam promoter cloning and construction of plasmids

A 2-kb fragment of the mouse Tfam promoter was generated by PCR using genomic DNA (Blackie and Hood, unpublished data). The mouse Tfam promoter was sequenced, matched to the GenBank sequence (Accession number AC153509) and used for PCR primer design (Table 1) to produce promoter truncations of 2051, 1706, 978, 393 and 78 bp from the transcription start site. These truncated constructs were gel extracted, digested with restriction enzymes and purified. They were then ligated into the XhoI and HindIII digested sites of the pGL3-basic vector containing the luciferase reporter gene.

**Table 1:** Primer sequences for mTfam constructs

Promoter constructs	
FRWD-2051	AAATACTCGAGGTAGCTGCAAATGCAGACAGT
FRWD-1706	AAATACTCGAGCTGGGCTTAGAAGGAGTGGG
FRWD-978	AAATACTCGAGTTGGCTGGCTAAGCTCATCT
FRWD-393	AAATACTCGAFCAGTAGCCTTGTGGGCTTTC
FRWD-79	AAATACTCGAGACAGCACCTCCTGTCCTCC
RVRSE 110	AAATAAAGCTTCGCTCGGGCCGACGAATGA
3'UTR Constructs	
FRWD-3'UTR	GACTCTAGAGATGGAAGACGGAGTT
Tfam 746	TGCGATATCCCACACAAGCTCTATG
Tfam 600	TGCGATATCCTATGCAAGAAAAACC

### Mouse Tfam 3′-UTR reporter constructs

Total RNA from C_2_C_12_ myotubes was extracted with TRIZOL reagent (Invitrogen) and RT was performed using Superscript III Reverse Transcriptase (Invitrogen) according to the manufacturer's instructions DNA fragments of 746 and 600 bp corresponding to the 3′-UTR of Tfam mRNA were generated by PCR from the reverse transcribed cDNA. These amplified 3′-UTR of Tfam fragments were gel extracted, digested with EcoRV and BamHI and then ligated into the HpaI and BamHI sites to replace the SV40 3′-UTR at the 3′-end of the Renilla Luciferase gene in the pRL (Renilla luciferase)-CMV (cytomegalovirus) vector.

### Transient transfection and promoter-luciferase analyses

C_2_C_12_ myoblasts were transfected and analysed as previously described [9]. Cells were cultured in six-well dishes and transiently transfected with 4 μg of each Tfam promoter-luciferase constructs using Lipofectamine 2000, following the manufacturer's instructions. The pGL3 empty vector was utilized to keep the amount of DNA transfected constant. Transfection efficiency was normalized to Renilla luciferase activity (pRL-CMV; 5 ng of transfected/well). Cell extracts were prepared on different days of differentiation using 1× passive lysis buffer. Luciferase activities were measured using an EG & G Berthold (Lumat LB9507) luminometer, according to the manufacturer's instructions.

### Tfam mRNA stability

Two methods were used to assess Tfam mRNA stability during differentiation. First, mRNA stability was assessed using the transfection of Tfam 3′-UTR constructs ligated to a Renilla luciferase reporter. C_2_C_12_ myoblast were transfected with 3.6 μg each of the control plasmids (pRL-CMV) or with 3.6 μg of Renilla luciferase/3′-UTR chimeras pTfam 746 or pTfam 600, as described above. Transfection efficiency was corrected using the 2051 kb Tfam promoter firefly luciferase construct (0.4 μg/well) and normalized to day 0 of Tfam promoter luciferase activity. Second, fully differentiated C_2_C_12_ myotubes were treated with either 10 μg/ml actinomycin D to inhibit transcription or methanol as a vehicle-matched control, for 2, 4 or 6 h. Total RNA was extracted with TRIZOL reagent (Invitrogen) according to manufacturer's instructions. The mRNA expression of mitochondrial transcription factor A (Tfam) over days of differentiation was quantified using 7300 StepOnePlus™ Real-time PCR System (Applied Biosystems Inc.) and SYBR® Green chemistry (PerfeC_T_a SYBR® Green Supermix, ROX, Quanta BioSciences). First-strand cDNA synthesis from 2 μg of total RNA was performed with primers using SuperscriptIII transcriptase (Invitrogen) according to manufacturer's directions. Forward and reverse primers (Tfam: Forward 5′-AAGGGAATGGGAAAGGTAGA-3′Reverse 5′-AACAGGACATGGAAAGCAGAT-3′; β-2 microglobulin: Forward 5′-GGTCTTTCTGGTGCTTGTCT-3′Reverse 5′-TATGTTCGGCTTCCCATTCT-3′) were designed based on sequences available in GenBank (http://www.ncbi.nlm.nih.gov/entrez/query.fcgi) using the MIT Primer 3 designer software (http://wi.mit.edu/cgi-bin/primer3/primer3_www.cgi) and were confirmed for specificity using the basic local alignment search tool (www.ncbi.nlm.nih.gov/BLAST/). β-2 Microglobulin was used as a control housekeeping gene, the expression of which did not differ between actinomycin D or vehicle treatment groups. All samples were run in duplicate simultaneously with negative controls that contained no cDNA. Melting point dissociation curves generated by the instrument were used to confirm the specificity of the amplified product. Primer efficiency curves were generated for each set to ensure 100±2% efficiency. Linear regression analysis of Tfam mRNA availability in actinomycin D-treated day 1 compared with day 4 differentiated C2C12 cells were plotted. The decay rates were determined by comparing the slopes of day 1 compared with day 4 of Tfam mRNA availability.

### mtDNA analyses

mtDNA and nuclear DNA (nDNA) were isolated with a Mammalian Genomic DNA Miniprep kit (Sigma–Aldrich). Forward and reverse primers for mtDNA (ND1 (NADH dehydrogenase, subunit 1): Forward 5′-CCTATCACCCTTGCCATCAT-3′/Reverse 5′-GAGGCTGTTGCTTGTGTGAC-3′); and nDNA (S12: Forward 5′-CGTCACCCGTGATTCACCGC-3′; Reverse 5′-CGCGTATGCCACGTGCTAGG-3′) were used. PCRs were conducted and the amplified products were analysed on Ethidium Bromide-stained agarose gels and expected size products were quantified using SigmaScan Pro (version 5) software. The quantification of all target genes was corrected using the internal control S12.

### Statistical analysis

Data are expressed as means±S.E.M. The luciferase activity graphs utilizing various Tfam promoter lengths for AICAR-treated cells and non-treated cells and Renilla luciferase/Tfam 3′-UTR chimera constructs were analysed using two-way ANOVAs followed by Bonferroni post-hoc tests. Students' unpaired *t*test was used to analyse the mtDNA and COX I mRNA data. All other experiments were analysed using a one-way ANOVA to examine the effect of differentiation. Data are reported significant if *P*<0.05.

## RESULTS

### Differentiation-induced changes in markers of mitochondrial content

Nuclear-encoded mitochondrial proteins were measured in C_2_C_12_ cells to assess changes in mitochondrial content during differentiation. COX IV (Figure 1A, *P*<0.05) and cytochrome *c* (Figure 1B, *P*<0.05) protein levels were significantly elevated by 3- and 2-fold respectively, on day 4 of differentiation compared with day 0. To further confirm that mitochondrial biogenesis occurred in C_2_C_12_ cells with differentiation, a functional marker of mitochondrial content was measured. Differentiation resulted in a significant 3-fold increase in COX activity by day 2 of differentiation, when compared with day 0 (Figure 1C, *P*<0.05).

**Figure 1:**
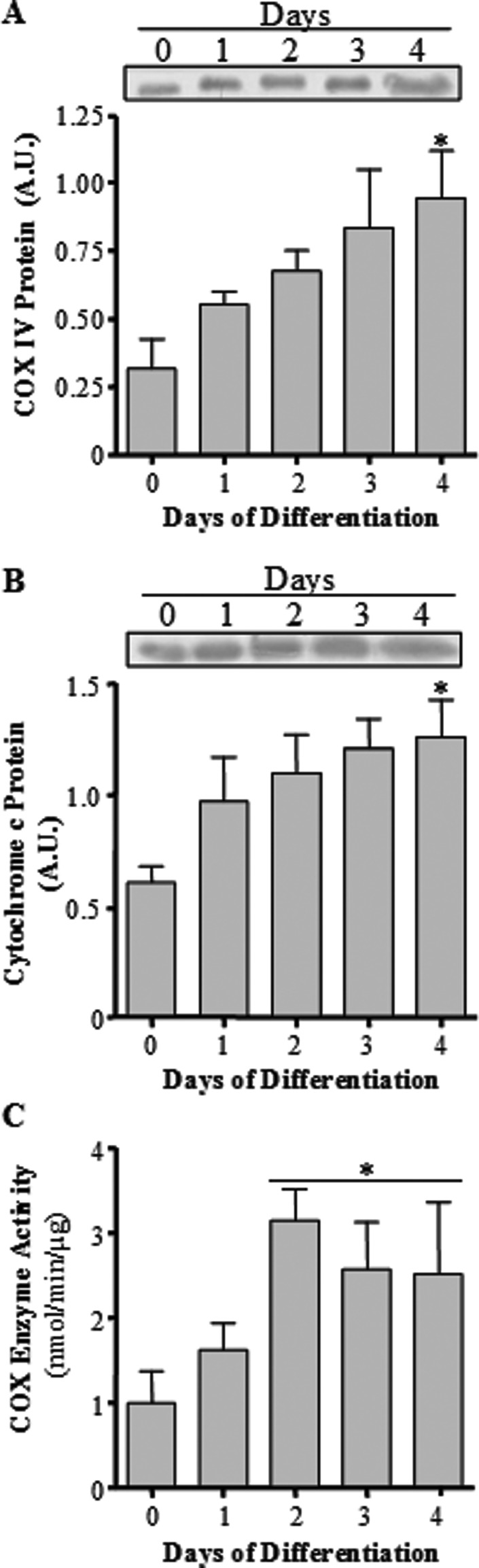
Differentiation-induced changes in markers of mitochondrial content Total extracts from C_2_C_12_ cells were isolated on each day of differentiation for 5 consecutive days. (**A**) COX IV (*n*=7). *P<0.05, day 4 compared with day 0. (**B**) cytochrome *c* (*n*=10). *P<0.05, day 4 compared with day 0. (**C**) COX activity (*n*=3) *P<0.05 day 2–4 compared with day 0. Representative blots are shown with the graphical quantification below. Values are expressed as means±S.E.M.

### Differentiation-induced changes in Tfam expression

Tfam transcription, mRNA and protein levels were analysed in relation to overall changes in mitochondrial content during differentiation. Tfam transcriptional activity increased by approximately 13-fold on day 2 of differentiation compared with day 0 and remained elevated by 30-fold on day 4 when compared with day 0 (Figure 2A, *P*<0.05). This occurred in concordance with an approximate 2-fold increase in Tfam mRNA levels (Figure 2B, *P*<0.05) on day 4 of differentiation. Additionally, differentiation produced an approximate 2-fold increase in Tfam protein level (Figure 2C, *P*<0.05) on day 4 of differentiation relative to myoblasts on day 0, which paralleled the induction of Tfam mRNA. Thus, significant increases in differentiation-induced transcriptional activation on day 2 preceded elevations in Tfam mRNA and protein expression evident by day 4.

**Figure 2:**
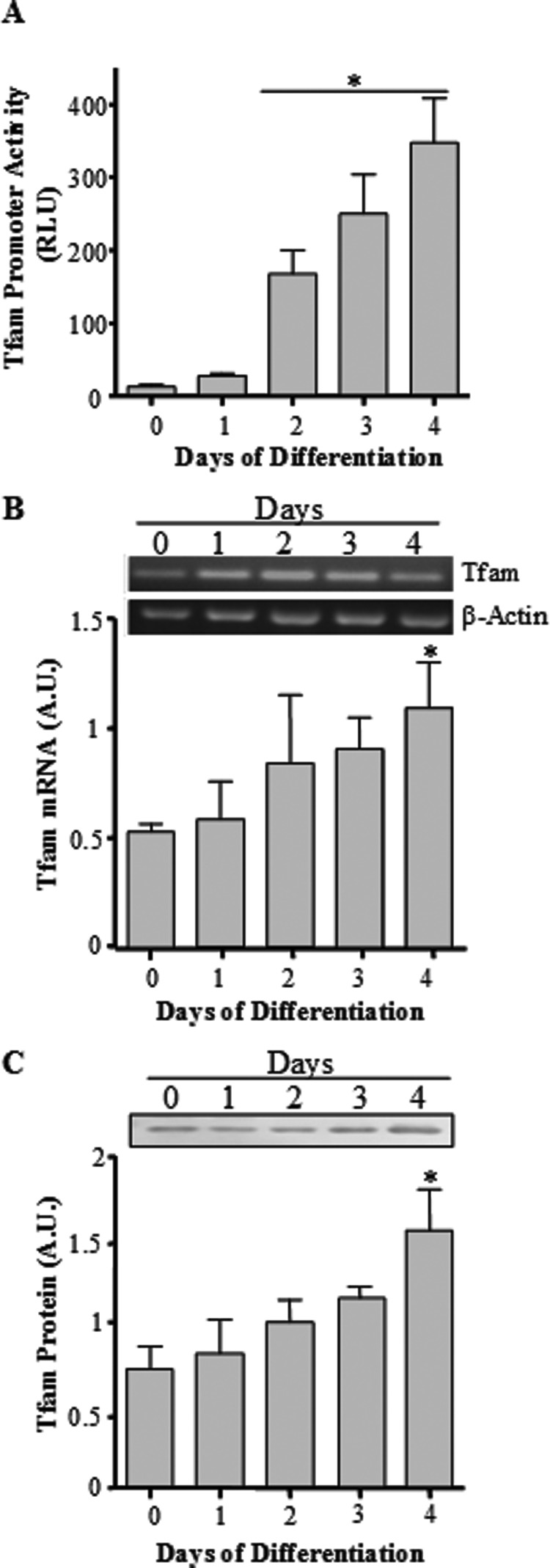
Differentiation-induced changes in Tfam expression (**A**) Relative luciferase activity of the full-length Tfam promoter (*n*=7). *P<0.05 day 2–4 compared with day 0. (**B**) Tfam mRNA (*n*=5). *P<0.05, day 4 compared with day 0. (**C**) Protein levels (*n*=6) *P<0.05, day 4 compared with day 0. Representative blots are shown with the graphical quantification below. Values are expressed as means±S.E.M.

### Differentiation-induced changes in Tfam localization

In order to localize Tfam during differentiation, mitochondrial and cytosolic fractions were isolated. At day 0, Tfam protein level was significantly higher in the cytosolic fraction (Figure 3, *p*<0.05). At days 2 and 4, Tfam accumulated within the mitochondria and was reduced in the cytosol. This indicates the progressive translocation of a greater abundance of Tfam into the mitochondrial fraction as differentiation proceeds.

**Figure 3:**
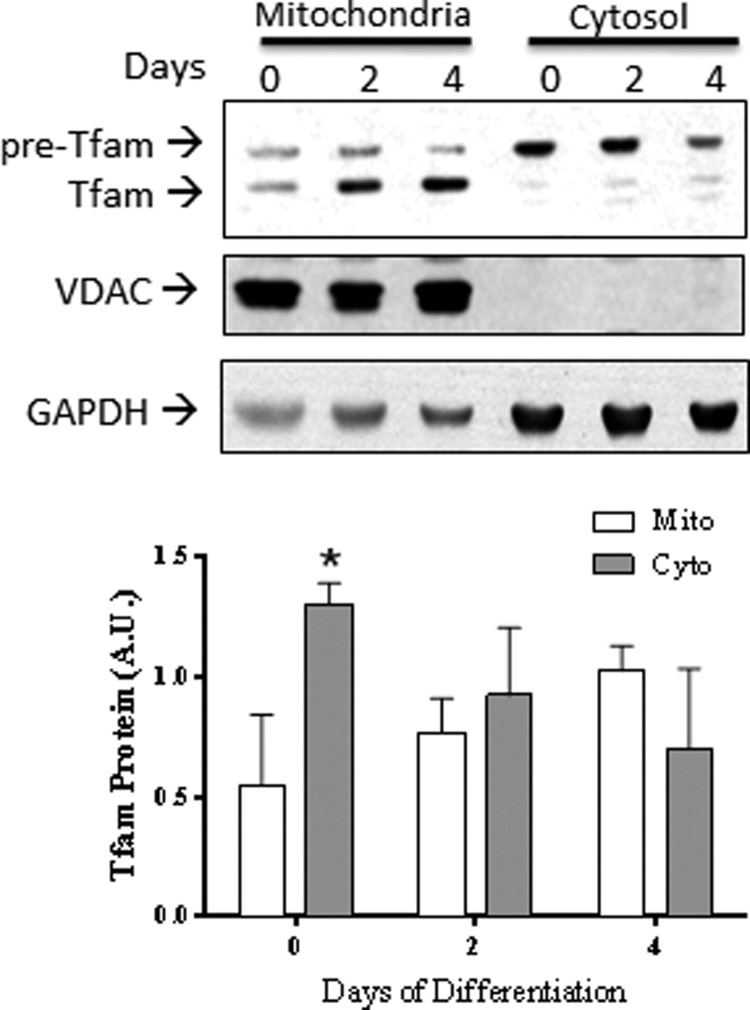
Differentiation-induced changes in Tfam localization Western blots (upper) and quantification (lower) of Tfam distribution in the mitochondria and cytosolic fractions at days 0, 2 and 4 days of differentiation. Tfam protein level was significantly higher in the cytoplasm (*P<0.05) at day 0 and then tended to decrease in cytoplasm and increase in the mitochondria fraction at days 2 and 4 of differentiation. VDAC and GAPDH are representative mitochondrial and cytosolic proteins respectively.

### Differentiation-induced changes in Tfam mRNA stability

To further investigate the differentiation-induced changes in Tfam expression, we analysed possible changes in Tfam mRNA stability. Knowing that the 3′-UTR of mRNAs is primarily responsible for the stability of mature mRNA, we examined this region of the Tfam mRNA. Analyses revealed the identification of several potential binding sites for either stabilizing (HuR) or destabilizing (CuGBP1, KSRP) RNA-binding proteins (Figure 4A), suggesting possible modulation of Tfam mRNA stability via these proteins, which are highly expressed in muscle. We then employed two different methods to assess mRNA stability. First, we inhibited transcription using actinomycin D at day 1 and day 4 of differentiation and examined the rate of decay of Tfam mRNA over a 6 h time course. Tfam mRNA was 3.2-fold more stable at day 4, compared with day 1 (Figure 4B). Second, we examined Tfam mRNA stability using long (746 bp) and shorter (600 bp) 3′-UTR-luciferase constructs at each day of differentiation. Luciferase measurements revealed no effect of differentiation on the activity of the 600 bp Tfam 3′-UTR. However, a significant increase in reporter activity was evident at both 3 and 4 days of differentiation when the longer 746 bp construct was assessed (Figure 4C). These data suggest that the increase in Tfam mRNA evident during differentiation is due, in part, to an increased stabilization of the Tfam mRNA transcript. Further, the data point to the distal 146 bp of the 3′-UTR as the main determining sequence of this increase in stability. Analysis of the sequence illustrates the presence of HuR-binding sites within this region, which could contribute to the greater stability with differentiation; however no changes in total cellular HuR, CuGBP1 or KSRP levels were observed during differentiation (Figure 4D). Indeed, the predominant localization of these RNA-binding proteins was in the nucleus, whereas considerably smaller amounts were found in the cytosol (Figure 5). This distribution remained unchanged with differentiation.

**Figure 4:**
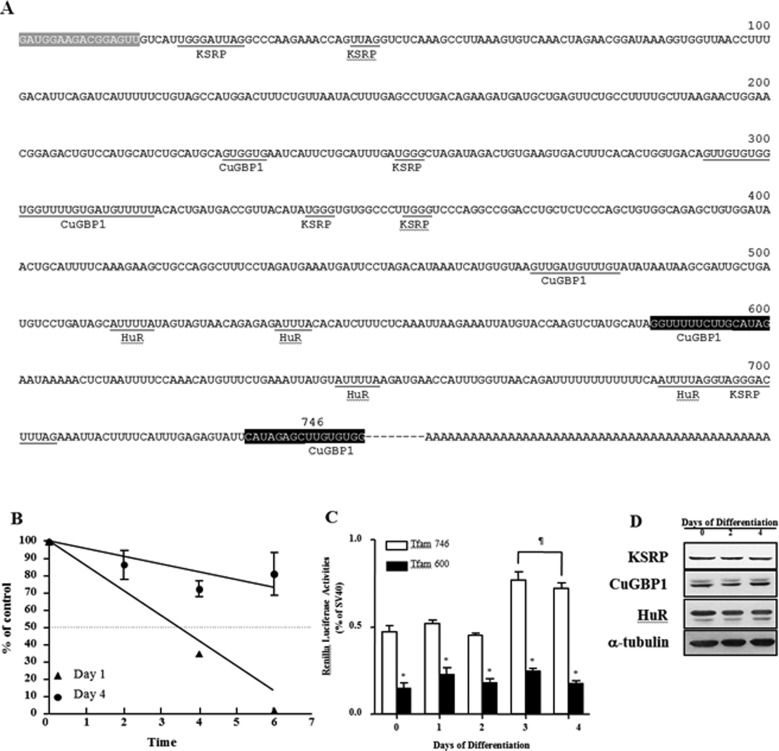
Differentiation-induced changes in Tfam mRNA stability (**A**) The nt sequence corresponding to the 3′-UTR of mouse Tfam is illustrated. Possible binding sites for RNA-binding proteins, HuR, CuGBP1 and KSRP are underlined. Primers used for 746 and 600 bp Tfam 3′-UTR constructs are highlighted in grey for the forward reaction and black for reverse reaction. (**B**) Linear regression analysis of Tfam mRNA availability in actinomycin D-treated day 1 compared with day 4 differentiated C_2_C_12_ cells (*n*=3). Slopes of day 1 (−14.45±2.657) and day 4 (−4.475±1.039) decay rates differentiated cells are 3.2-fold different (*n*=3 experiments). (**C**) Effect of differentiation on Renilla luciferase activities of 746 and 600 bp Tfam 3′-UTRs. (Day 3–4 compared with day 0–2; *n*=4, ^¶^*P*<0.001; 746 compared with 600 bp Tfam 3′-UTR; *n*=4, *P<0.001). Values are expressed as means±S.E.M. (**D**) Total cellular protein levels of HuR, CuGBP1 and KSRP at days 0, 2 and 4 of differentiation. Representative blots of seven experiments are shown.

**Figure 5:**
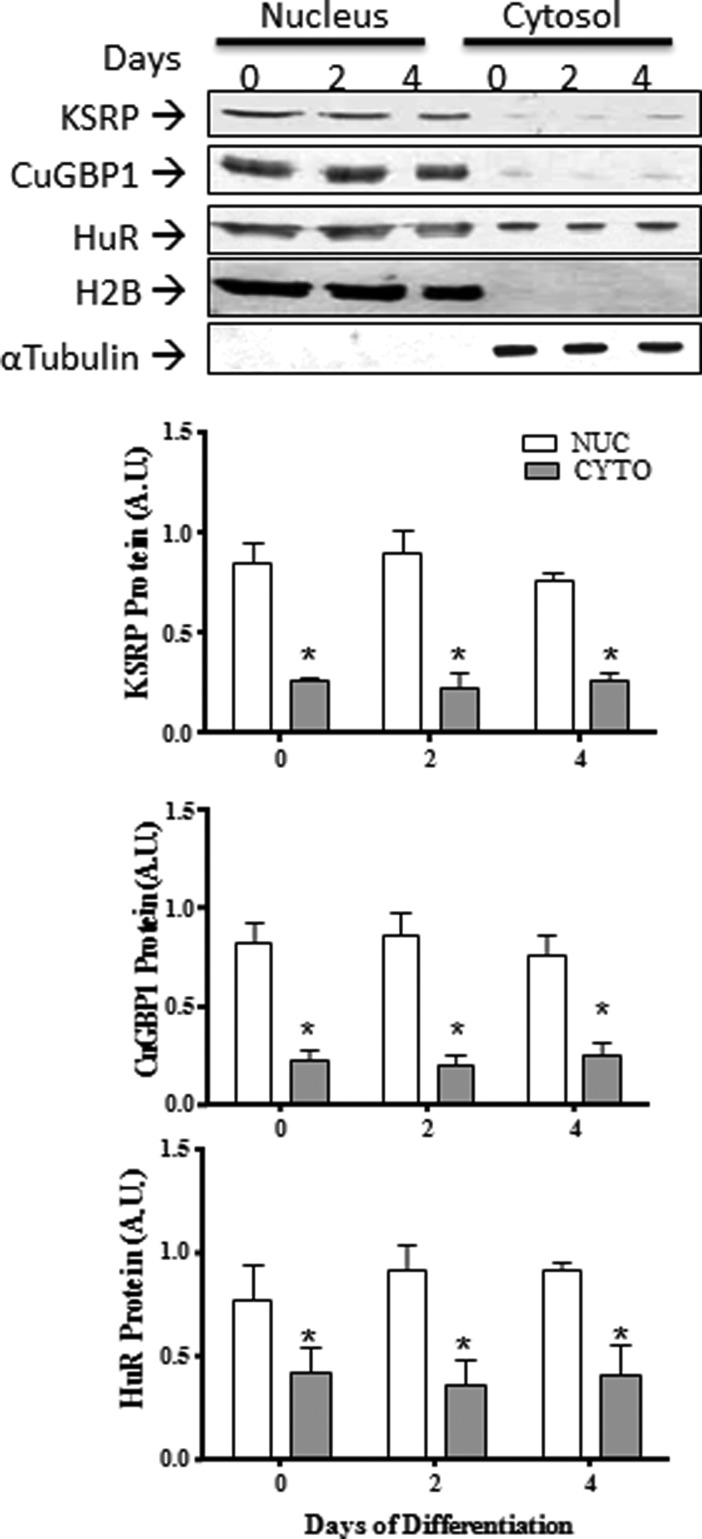
Distribution of RNA-binding proteins during differen-tiation RNA-binding proteins were measured in nuclear and cytosolic fractions at 0, 2 and 4 days of differentiation. Western blots (upper) were quantified (lower) revealing a significant difference between the levels of each protein in the cytosol compared with the nuclear fractions (*P<0.05). H2B and tubulin are representative nuclear and cytosolic proteins respectively.

### Expression of downstream targets of Tfam in response to differentiation

To assess the functional nature of the increase in Tfam protein during differentiation, we measured downstream targets of Tfam transcription. COX I, an mtDNA-encoded protein subunit of complex IV, was elevated by approximately 2-fold (Figure 6A, *P*<0.05) on day 4 of differentiation when compared with day 0. Furthermore, mtDNA copy number increased by 1.5-fold (Figure 6B, *P*<0.05) in fully formed myotubes on day 4 in contrast with the day 0 myoblasts.

**Figure 6:**
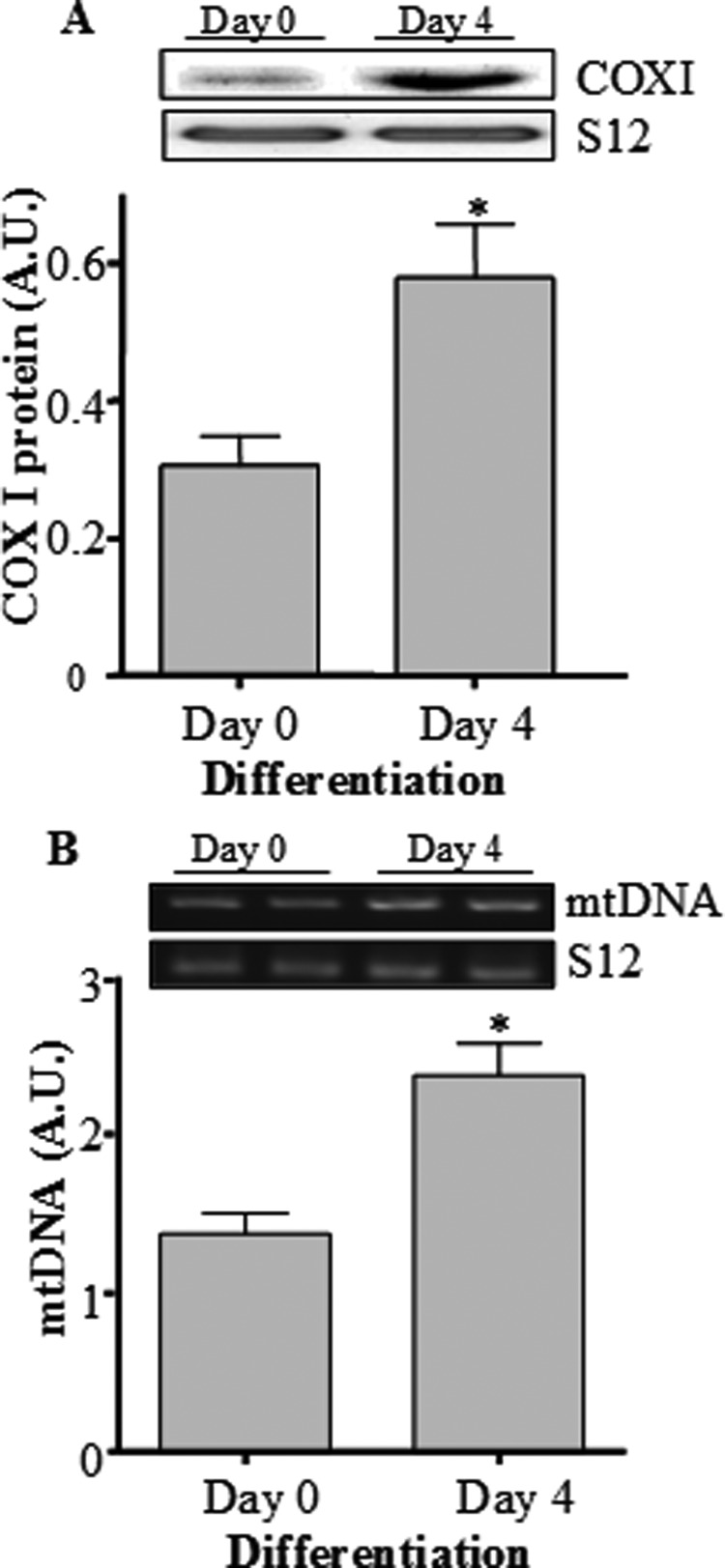
Expression of downstream targets of Tfam in response to differentiation (**A**) Protein level of mitochondrial-encoded COX subunit I of the electron transport chain (*n*=3) and (**B**) mtDNA copy-number in myoblasts of C_2_C_12_ muscle cells compared with day 4 myotubes (*n*=3). Representative blots are shown with the graphical quantification below. Values are expressed as means±S.E.M. *P<0.05, day 4 compared with day 0.

### Characterization of the 2 kb mouse Tfam promoter

A thorough examination of the mouse Tfam promoter sequence up to −2051 bp upstream of the transcription start site was conducted to investigate the location of putative binding sites for transcription factors involved in regulating Tfam transcription during differentiation. The promoter sequence was retrieved from Transcriptional Regulatory Element Database (Accession Number 48715) and the sequence was subjected to analysis for the presence of consensus transcription factor-binding sites that were 6–8 nts long using PATCH software. A number of binding sites of known transcription factors involved in mitochondrial biogenesis were identified (Figure 7).

**Figure 7:**
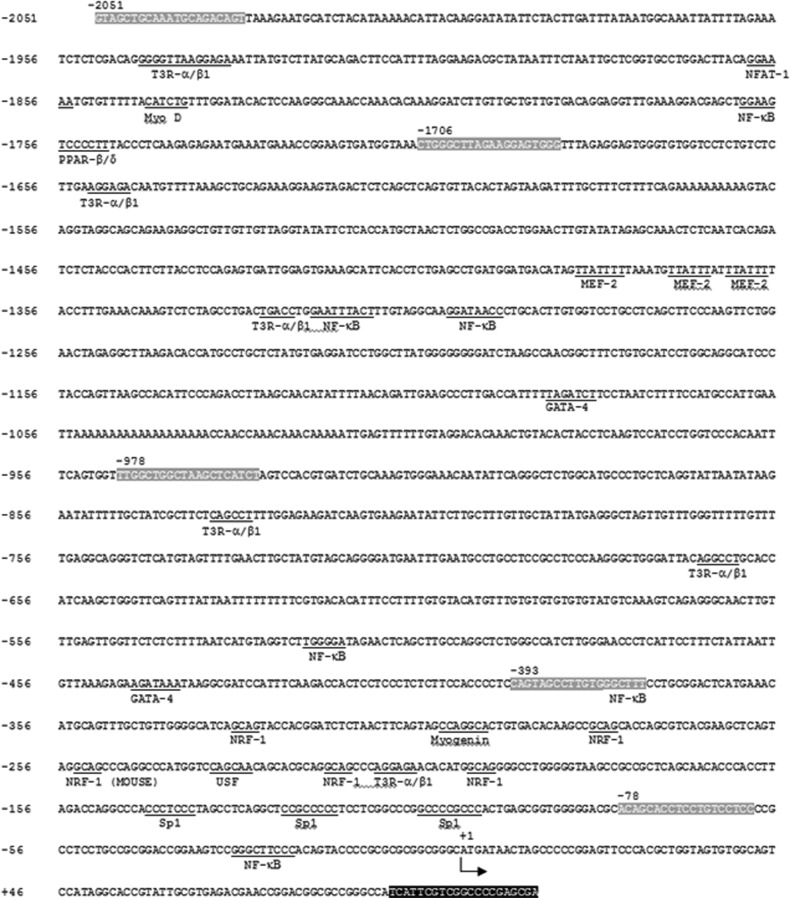
Mouse Tfam promoter The nt sequence from +110 to −2051 bp of the mouse Tfam promoter is illustrated. The arrow indicates the transcription start site. Possible binding sites for transcription factors are underlined and specified. Primers used for truncation of the promoter are highlighted in grey for the forward reaction and in black for the reverse reaction.

### Promoter activity of various mouse Tfam truncations

To investigate possible differences in the functions of the regions within the 2 kb Tfam promoter, 5′-deletion constructs were transfected into C_2_C_12_ cells to determine transcriptional activity. The data indicate that Tfam transcription increased with each day of differentiation for all promoter truncations investigated. The activity of the 1706 construct was 4.5-fold higher, on average, than the full-length 2051 bp construct from days 2 to 4 of differentiation (Figure 8, *P*<0.05), indicating the presence of a major repressor element(s) between 1706 and 2051 bp upstream of the start site. Further truncation of the promoter produced progressive decreases in promoter transcriptional activity, such that the activity of the 78 bp construct reporter activity was 50% lower (Figure 8, *P*<0.05) than the 1706 mTfam construct during successive days of differentiation.

**Figure 8:**
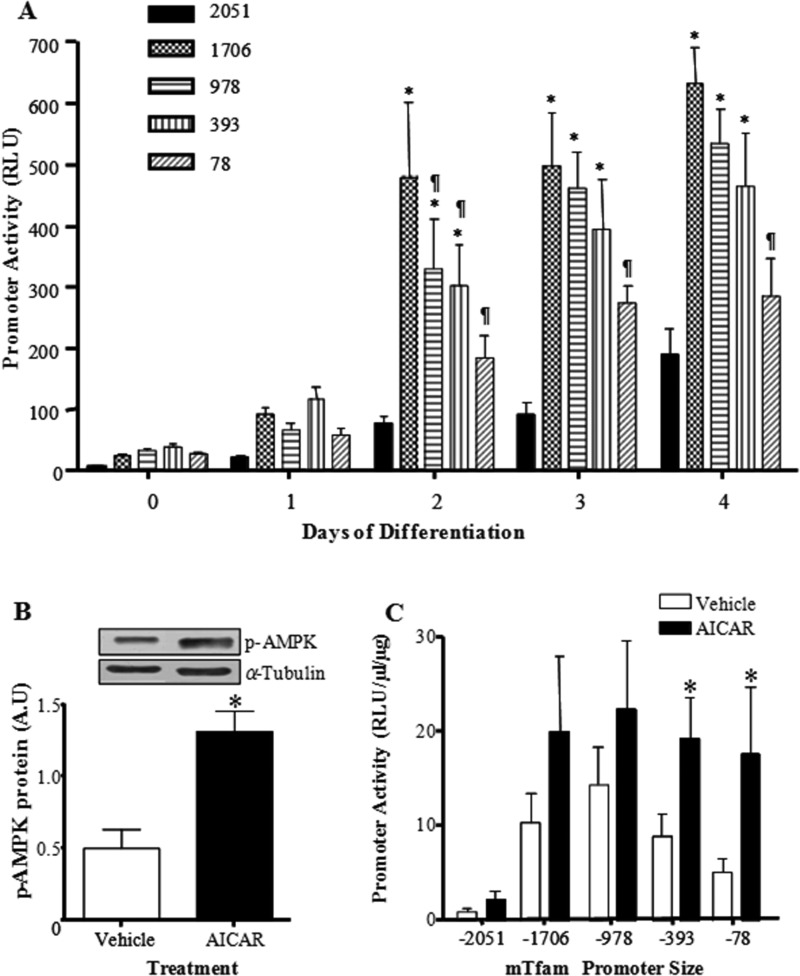
Transcriptional activity of Tfam promoter truncations and effect of AMPK activation (**A**) The responsiveness of the various promoter-luciferase reporter constructs to differentiation-induced activation was assessed on days 0, 1, 2, 3 and 4 (*n*=7). *P<0.05, −1706 compared with −2051 kb, −978 kb compared with −2051 kb, −393 compared with −2051 kb. ^¶^*P*<0.05, −978 kb compared with −1706 kb, −393 kb compared with −1706 kb, −78 kb compared with −1706 kb. (**B**) C_2_C_12_ cells were treated with AICAR (1 mM) or vehicle for 24 h. Representative western blot probed with Phospho-AMPKα (Thr^172^). Summary of the effect of AICAR on AMPK activation is shown (*n*=3). (**C**) The relative luciferase activity of the Tfam promoter constructs in vehicle- or AICAR-treated cells (*n*=7). *P<0.05, AICAR compared with vehicle. Values are expressed as means±S.E.M.

### Effect of AICAR on AMPK activation and Tfam transcription

AICAR was utilized to investigate the possible role of AMPK in the transcriptional control of Tfam expression in C_2_C_12_ cells. Following treatment with AICAR, AMPK activation via phosphorylation on Thr^172^ was increased by 2.5-fold (Figure 8B, *P*<0.05). In response to AMPK activation, Tfam promoter activity was increased by approximately 2-fold when transfected with the −393 and −78 kb Tfam promoter constructs (Figure 8C, *P*<0.05).

### Expression of possible transcription factors affecting the Tfam promoter during differentiation

Of the multitude of putative binding sites (Figure 7) within the Tfam promoter, Sp1 and NF-κB (nuclear factor κB; represented by the p65 subunit) were measured during differentiation since they have been previously implicated in both the regulation of mitochondrial content and the myogenesis [13–16]. NF-κB-p65 was negatively regulated during differentiation, producing a gradual reduction in protein level at day 4 to 55% of the level found at day 0 (Figure 9A, *P*<0.05). In contrast, Sp1 (Figure 9B, *P*<0.05), a transcriptional regulator of a variety of mitochondrial proteins, remained unchanged during differentiation.

**Figure 9:**
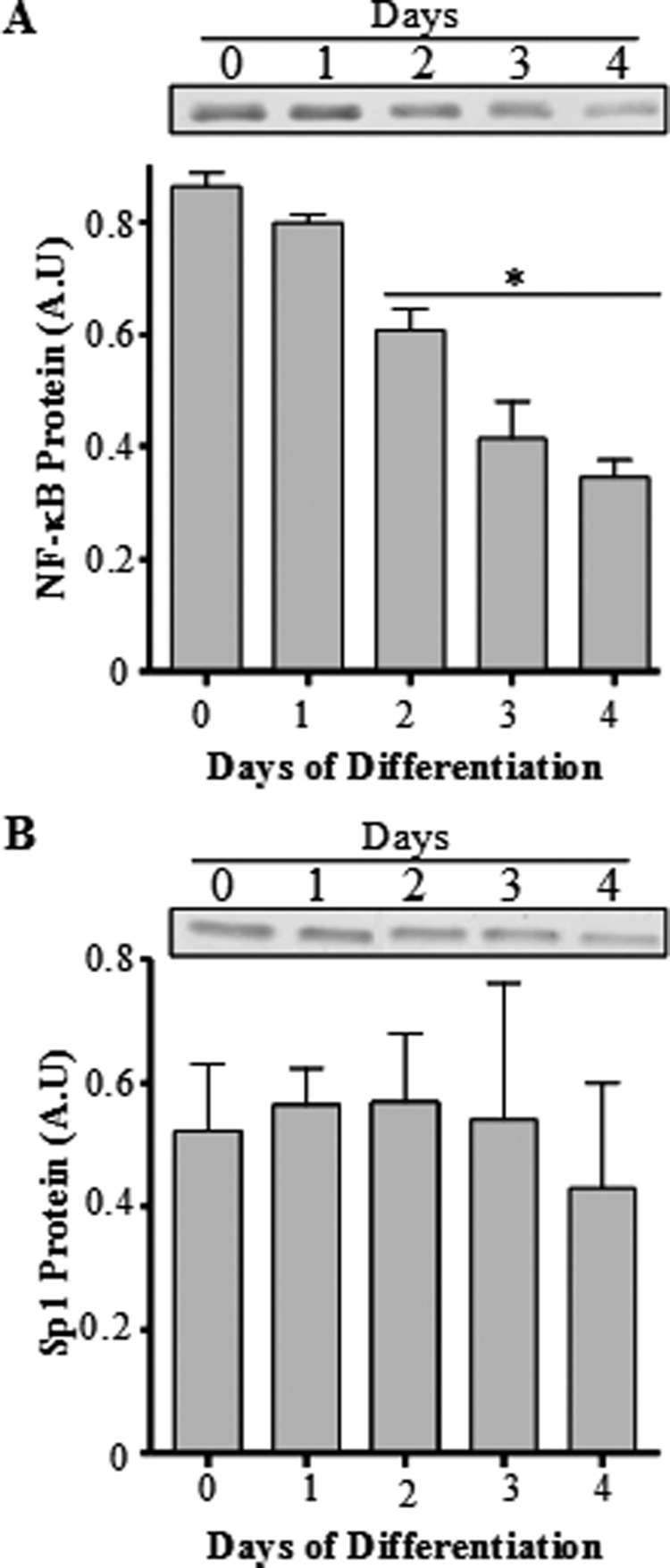
Nuclear transcription factors of genes important for mitochondrial biogenesis (**A**) NFκB p65 (*n*=3) and (**B**) Sp1 (*n*=6) protein levels in response to 4 days of differentiation. Representative blots are shown with the graphical quantification below. Values are expressed as means±S.E.M. *P<0.05, day 2–4 compared with day 0.

## DISCUSSION

The differentiation of myoblasts into myotubes is accompanied by a pronounced rise in mitochondrial content [1], which is a process highly dependent on the intricate co-operation of both the nuclear and the mitochondrial genomes. Our data show that differentiation is accompanied by the rapid augmentation of established markers of mitochondrial biogenesis, including COX, presumably to facilitate aerobic energy supply for the process of myogenesis. Since COX is a multi-subunit holoenzyme containing components derived from the expression of mtDNA, it is likely that this increase is mediated, in part, by changes in Tfam expression and activity. Tfam is a nuclear-encoded mitochondrial transcription factor which allows for the nuclear control of mtDNA gene transcription and replication. Disruption of the Tfam gene in the mouse germ line using a cre-lox P recombinant system depleted mtDNA copy number in heart [17], liver [7], kidney [7] and skeletal muscle cells [18], creating severe respiratory chain deficiencies. In contrast, endurance exercise-induced stimulation of mitochondrial biogenesis enhances Tfam expression and subsequently increases mitochondrial transcripts and function [19,20]. Thus, our overall understanding of mitochondrial biogenesis in skeletal muscle would benefit from an elucidation of the regulation of Tfam expression, from the level of transcription, to its functional consequences within the mitochondrial matrix.

We began to investigate this by establishing how differentiation affects the expression of Tfam. Our results demonstrate enhanced transcriptional activity of the Tfam promoter on day 2 of differentiation, with subsequent increases in Tfam mRNA levels observed on day 4. Changes in the transcriptional activity of the Tfam promoter exceeded the increase in steady-state mRNA levels. This suggested the likelihood of rapid Tfam mRNA decay at the onset of the differentiation period to counterbalance this large increase in transcription. Indeed, Tfam mRNA degradation was much more rapid at the onset of differentiation (day 1), as compared with day 4. In contrast with this complex interaction between transcription (synthesis) and degradation leading to steady state Tfam mRNA levels, the progressive elevation in Tfam protein levels observed over the differentiation period paralleled that of the mRNA levels. This suggests a simpler model for the regulated expression of Tfam protein, which is controlled by the level of mRNA, with little regulation evident at the level of translation.

The specific targeting sequence within the N-terminal of the Tfam dictates the import of the protein into the mitochondria, where it can carry out its functional role on mtDNA [6]. Cellular fractionation experiments confirmed the progressive accumulation of Tfam within the organelle and a reduction in Tfam within the cytoplasm, suggesting an increase in the import process during differentiation. Once imported, Tfam can promote the transcription and replication of the mitochondrial genome, leading to increased mtDNA copy number and increased transcription of its 13 protein gene products. After 4 days of differentiation, both mtDNA copy number and expression of COX subunit I were markedly elevated in C_2_C_12_ myotubes in proportion to each other. These data suggest that the observed increase in mitochondrial Tfam protein during differentiation is functional and that following translocation into the mitochondria, its predominant influence is on the rate of mtDNA replication, rather than transcription. Thus, adaptations in the Tfam expression pathway are a normal component of the adaptive responses of the organelle during mitochondrial biogenesis induced by differentiation. Our previous results in mature adult skeletal muscle also showed that contractile activity-induced elevations in Tfam protein levels permitted greater Tfam binding within the D-loop of mtDNA and that this corresponded to greater expression of mitochondrial-encoded COX subunit III transcript [20]. This indicates that Tfam contributes to mitochondrial biogenesis as induced by markedly different stimuli, including differentiation and contractile activity.

Other studies have shown that the transcriptional rate of the Tfam gene appears to be regulated, in part, by nuclear respiratory factors and Sp1 [16]. Motif mutation studies in rats have indicated that nuclear respiratory factor 1 (NRF-1), NRF-2 and Sp1 promote basal Tfam gene transcription. However, other important regulatory sequences within the promoter involved in the activation of the gene during differentiation remain to be determined. Therefore, we analysed the 2 kb upstream promoter fragment to identify candidate transcription factor-binding sites that could mediate gene activation during differentiation. Our search identified sites for proteins which regulate mitochondrial metabolism (PPAR (peroxisome proliferator-activated receptor), T3R), muscle development (MEF-2 (myocyte enhancer factor 2), myogenin, MyoD) and AMPK-targeted transcription factors (USF (upstream transcription factor) and GATA-4 (GATA binding protein 4)). Additionally, this 2 kb sequence contains putative recognition sites for a variety of transcriptional activators that are commonly found within the promoters of nuclear-encoded mitochondrial genes. Thus, we generated a series of 5′-deletion constructs to further identify the regulatory regions responsible for activation of the promoter during differentiation. Interestingly, promoter activity increased with all truncations of the 2-kb promoter, including the minimal 78-bp construct, with differentiation. This suggests that, by 4 days of differentiation, the activity of this promoter fragment along with the greater mRNA stability observed at this time point, are sufficient to drive the synthesis of Tfam mRNA and thus Tfam protein. Future work could therefore be directed at elucidating the regulatory elements and binding proteins within this segment which provide at least as much transcriptional activity as the much larger, 2051 bp promoter. The intermediate constructs, particularly the 1706 fragment, show a remarkable increase in activity between 1 and 2 days of differentiation which is much higher than the larger 2 kb fragment. This indicates the presence of fairly dominant silencing elements between −1706 and −2051 upstream of the transcription start site. A possible candidate for this includes NF-κB, a negative myogenic transcription factor [15,21]. The NF-κB activation pathway is mediated by inhibitor of κB kinase (IKK)β and IKKγ, leading to the activation of NF-κB/p65 that binds DNA and regulates gene expression to inhibit myogenesis. It was previously shown that NF-κB/p65 binds and inhibits the transcription of MyoD, thereby inhibiting the progression of muscle differentiation [14,15]. Thus, it is possible that the binding of NF-κB to its putative element at −1761 bp within the mouse Tfam promoter, confers the same response on the Tfam promoter, suppressing the transcription of the Tfam gene. The reduction in NF-κB expression observed with differentiation could allow for the expression of MyoD and thereby promote muscle differentiation. The elevation in Tfam reporter activity observed with early differentiation may be attributed to the reduction in NF-κB protein levels observed in the mature myotubes. In support of this, removal of the first 345 bp of the Tfam promoter, which encompasses the NF-κB-binding site, resulted in a large induction of Tfam-luciferase activity. Furthermore, the increase in Tfam promoter activity observed between −78 bp and the −1706bp construct indicates the presence of binding sites for potent transcriptional activators in this region. The NRF-1 and Sp1 consensus sites, found at −254 and −145 respectively [16], are plausible important candidates that likely contribute to promoter activation during differentiation. However, removal of these sites with the −78 construct did not result in full ablation of promoter activity, indicating the presence of alternative transcription factors which are important for Tfam transcriptional activation. Additional studies using ChIP analyses will be essential in future experiments to unravel the important transcription factors involved in Tfam transcription during differentiation.

Several studies have established that the expression of a variety of mitochondrial genes is increased via the activation of multiple serine-threonine kinases in skeletal muscle. This often occurs through alterations in the energy status of the cell that activate AMPK as a result of increased AMP:ATP ratios [22]. Activation of AMPK with the use of the AMP-analogue AICAR has been associated with elevations in the expression of PGC-1α, mitochondrial proteins and higher mitochondrial enzyme activities [8,9,23]. However, the detailed molecular mechanisms, as well as the direct effect on Tfam gene transcription, have yet to be established. Thus, we employed AICAR treatment to investigate the involvement of AMPK in Tfam gene expression. Our data illustrate that AICAR-positive regions exist in the most proximal region of the Tfam promoter, whereas other regions are not significantly affected by the treatment. This is identified by the 2- and 3-fold induction in Tfam reporter activity with AICAR treatment for the −393 and −78 constructs respectively, suggesting that AMPK may target transcription factors that bind to the Tfam promoter within this region. AICAR also appears to affect the binding of transcriptional activators to the Tfam promoter between −78 bp and the transcription start site; however, the identity of these binding proteins remains unknown.

Differing models of mitochondrial biogenesis produce similar adaptive responses in mitochondrial proteins [24] and transcription factors, such as Sp1 [13]. The characterization of Sp1 as an important basal regulator of Tfam expression [16] prompted further exploration into the regulatory protein. Surprisingly, despite the rapid increase in mitochondrial content produced by differentiation, no effect on Sp1 protein levels was observed. This suggests that either (1) the functional role of Sp1 is not dependent on variations in the level of the protein, but rather on the activity of the protein itself or (2) Sp1 does not play a role in mediating Tfam transcription during muscle cell differentiation, as it does in other cell types.

In summary, we have investigated the regulation Tfam expression in muscle cells during differentiation, which is accompanied by a marked increase in mitochondrial biogenesis. Our results suggest that the increase in total cellular Tfam protein is regulated by a combination of transcriptional activation, as well as by changes in mRNA stability, with little regulation at the level of translation. A negative regulatory element within the distal promoter region probably attenuates transcription and therefore overall protein expression, within muscle cells. In turn, this transcriptional activation is probably influenced by the energy state of the cell during differentiation, which affects the activity of AMPK. The import of Tfam to mitochondria may represent an additional site of Tfam activity regulation, but this remains to be specifically determined. However, it is evident that as it becomes progressively localized to the organelle matrix, Tfam becomes active in the replication of mtDNA and the generation of mtDNA gene products which contribute to the co-ordinated regulation of organelle biogenesis.

## Abbreviations

AMPK: AMP-activated protein kinase
COX: cytochrome *c* oxidase
DMEM: Dulbecco's modified Eagle's medium
GAPDH: glyceraldehyde-3-phosphate dehydrogenase
IKK: inhibitor of κB kinase
nDNA: nuclear DNA
NF-κB: nuclear factor κB
P/S: penicillin/streptomycin
RT: reverse transcription
Tfam: mitochondrial transcription factor A
TFB1M: mitochondrial transcription factor B1
